# Phase I clinical trial of the base excision repair inhibitor methoxyamine in combination with fludarabine for patients with advanced hematologic malignancies

**DOI:** 10.18632/oncotarget.20094

**Published:** 2017-08-09

**Authors:** Paolo F. Caimi, Brenda W. Cooper, Basem M. William, Afshin Dowlati, Paul M. Barr, Pingfu Fu, John Pink, Yan Xu, Hillard M. Lazarus, Marcos de Lima, Stanton L. Gerson

**Affiliations:** ^1^ Case Comprehensive Cancer Center, Cleveland, Ohio, USA; ^2^ Division of Hematology and Oncology, Department of Medicine, University Hospitals Seidman Cancer Center, Cleveland, Ohio, USA; ^3^ Division of Hematology. The Ohio State University Medical School, Columbus, Ohio, USA; ^4^ Division of Hematology and Oncology, Department of Medicine, University of Rochester, Rochester, New York, USA; ^5^ Department of Biostatistics, Case Western Reserve University, Cleveland, Ohio, USA

**Keywords:** phase I, methoxyamine, base excision repair, hematologic malignancies, pharmacokinetics

## Abstract

**Purpose:**

We determined the safety, pharmacokinetics, pharmacodynamics and recommended phase II dose of the base excision repair blocker methoxyamine combined with fludarabine.

**Materials and Methods:**

This was a phase I study with intravenous fludarabine (25 mg/m^2^, days 1–5), and methoxyamine (15 mg/m^2^–120 mg/m^2^, once). A maximum of six cycles were given. Adult patients with relapsed/refractory hematologic malignancies, excluding acute myeloid leukemia, were eligible.

**Results:**

Twenty patients were treated; diagnoses included CLL/SLL (*n* = 10), follicular lymphoma (*n* = 3), DLBCL (*n* = 3), mantle cell lymphoma (*n* = 1), anaplastic large cell lymphoma (*n* = 1) and plasma cell myeloma (*n* = 2). No DLTs were observed and dose escalation reached the maximum planned dose. Hematologic toxicity was frequent; most common grade 3–4 toxicities were lymphopenia (70%), neutropenia (60%), leukopenia (50%) and anemia (40%). Four patients achieved a partial remission and 8 achieved stable disease. The drug combination resulted in increased DNA damage measured with the Comet assay.

**Conclusions:**

Methoxyamine combined with fludarabine was safe and well tolerated. Hematologic toxicity was comparable to single agent fludarabine. Activity appears to correlate with increased levels of DNA damage. Further studies will examine use of this combination of as part conditioning regimens of stem cell transplant and use of methoxyamine as fludarabine dose-sparing agent.

## INTRODUCTION

The purine analog fludarabine is most commonly used in the treatment of chronic lymphocytic leukemia (CLL) [1, 2] but is also part of the therapeutic armamentarium for indolent lymphomas [3, 4] and myeloid malignancies [5]. In CLL, fludarabine alone achieves superior response rates and progression free survival (PFS) than alkylating agents, albeit without overall survival improvement (OS) [1, 6]. Fludarabine is also an important component of reduced intensity conditioning regimens for allogeneic stem cell transplant [7].

The active metabolite fludarabine triphosphate (F-ara-ATP) exerts its cytotoxic activity through incorporation into DNA. In replicating cells, F-ara-ATP forms abnormal base pairs with thymine (F:T), interferes with DNA chain elongation by DNA polymerase α [8] and inhibits several other enzymes involved in DNA synthesis [9, 10]. In resting cells, F-ara-ATP is incorporated into DNA by the nucleotide excision repair pathway (NER) and terminates the DNA polymerization stage of the NER process [11, 12]. The resulting DNA damage leads to cell death, mediated either by p53 [13, 14] or PARP [10].

The base excision repair pathway (BER) is a DNA repair mechanism distinct from NER, capable of recognizing single base pair abnormalities [15], including those caused by exposure to environmental mutagens and antineoplastic agents [16–19]. The initial step in BER is removal of the abnormal base by a specific DNA glycosylase, forming an apurinic/apyrimidinic (AP) site. The AP site is cleaved by AP endonuclease (APE), generating a single - strand DNA break, followed by incorporation of the correct base and subsequent annealing by DNA polymerase β and DNA ligase, respectively [20]. The activity of the BER system confers resistance against antineoplastic agents that cause DNA damage [21]. More importantly, BER inhibition augments the cytotoxic activity of DNA damaging agents [21–23].

Methoxyamine is a small organic amine that inhibits BER by reacting in a specific fashion with the aldehyde group in the sugar moiety present at the AP site. Methoxyamine-bound AP sites are refractory to the lyase activity of APE, block further actions of BER pathway and result in persistent DNA lesions and arrested DNA replication, causing additional DNA damage by acting as topoisomerase IIα poisons [24]. Methoxyamine enhances the *in vitro* and *in vivo* activity of alkylating agents in colon, ovarian and breast cancer cell lines and xenograft models [25–29]. In a previous report, the addition of methoxyamine to fludarabine resulted in increased markers of DNA double strand breaks in cell lines and primary CLL cells, including increased Comet tail length and increased γH2AX expression, and result in induction of topoisomerase II expression [24]. In contrast, methoxyamine alone does not result in DNA double strand breaks or increased apoptosis compared to untreated controls [24, 30]. In mouse xenograft studies, methoxyamine augmented the antitumor activity of fludarabine, while methoxyamine alone did not have an antineoplastic effect [24].

Based on this preclinical data, we conducted a phase I study investigating the safety and tolerability of the combination of methoxyamine and fludarabine in the treatment of advanced hematologic malignancies.

## RESULTS

### Patient characteristics

Twenty patients were enrolled and treated in this clinical trial. Median age was 64 years (range 45 to 82); 60% of patients were men. The diagnoses included CLL/small lymphocytic lymphoma (SLL) (*n =* 10) follicular lymphoma (*n =* 3), diffuse large B cell lymphoma (DLBCL) (*n =* 3), mantle cell lymphoma (*n =* 1) and plasma cell myeloma (*n =* 2). The median number of prior lines of therapy was 3 (range 1 – 5) and 8 out of 10 CLL/SLL patients had received fludarabine, with 2 patients considered fludarabine refractory, relapsing within 6 months after a fludarabine – containing regimen (Table 1). All patients who completed at least one cycle of fludarabine and methoxyamine were considered evaluable for safety.

**Table 1:** Patient demographic and clinical characteristics

Characteristic	No. of patients
**Patients who received at least one cycle of fludarabine and methoxyamine**	20
**Age, years**	
Median	64
Range	45–82
**Sex**	
Male	12 (60%)
Female	8 (40%)
**Number of prior treatments**	
1 – 2	7 (35%)
≥ 3	13 (65%)
**Prior treatment with fludarabine**	8 (40%)
**Diagnoses**	
CLL/SLL	10 (50%)
Follicular lymphoma	3 (15%)
Diffuse large B cell lymphoma	3 (15%)
Mantle cell lymphoma	1 (5%)
Plasma cell myeloma	2 (10%)
**Eastern Cooperative Oncology Status**	
0	4 (20%)
1	15 (75%)
2	1 (5%)

### Treatment

In cycle 1, patients received fludarabine at a dose of 25 mg/m^2^, intravenously over 1 hour on days 1–5 of each cycle. Methoxyamine was infused on day 2 at the assigned dose level as intravenous infusion over 30 minutes, before fludarabine infusion. In cycle 2 and subsequent cycles, methoxyamine was given on day 1 of each cycle, before the infusion of fludarabine. The median number of cycles administered was 3 (range, 1 to 6). Seventeen patients discontinued treatment before completing the planned 6 cycles of therapy. The reasons for discontinuing therapy included disease progression (*n =* 7, 35%), adverse events (AEs) (*n =* 4, 25%: grade 2 fatigue [*n* = 2]; grade 3 pulmonary infection [*n* = 1] and prolonged neutropenia [*n* = 1]); patient choice (*n =* 4, 20%), continuation to allogeneic stem cell transplant (*n =* 2, 10%). Three patients completed the full plan of six cycles of treatment.

### Pharmacokinetics

**Figure 1:**
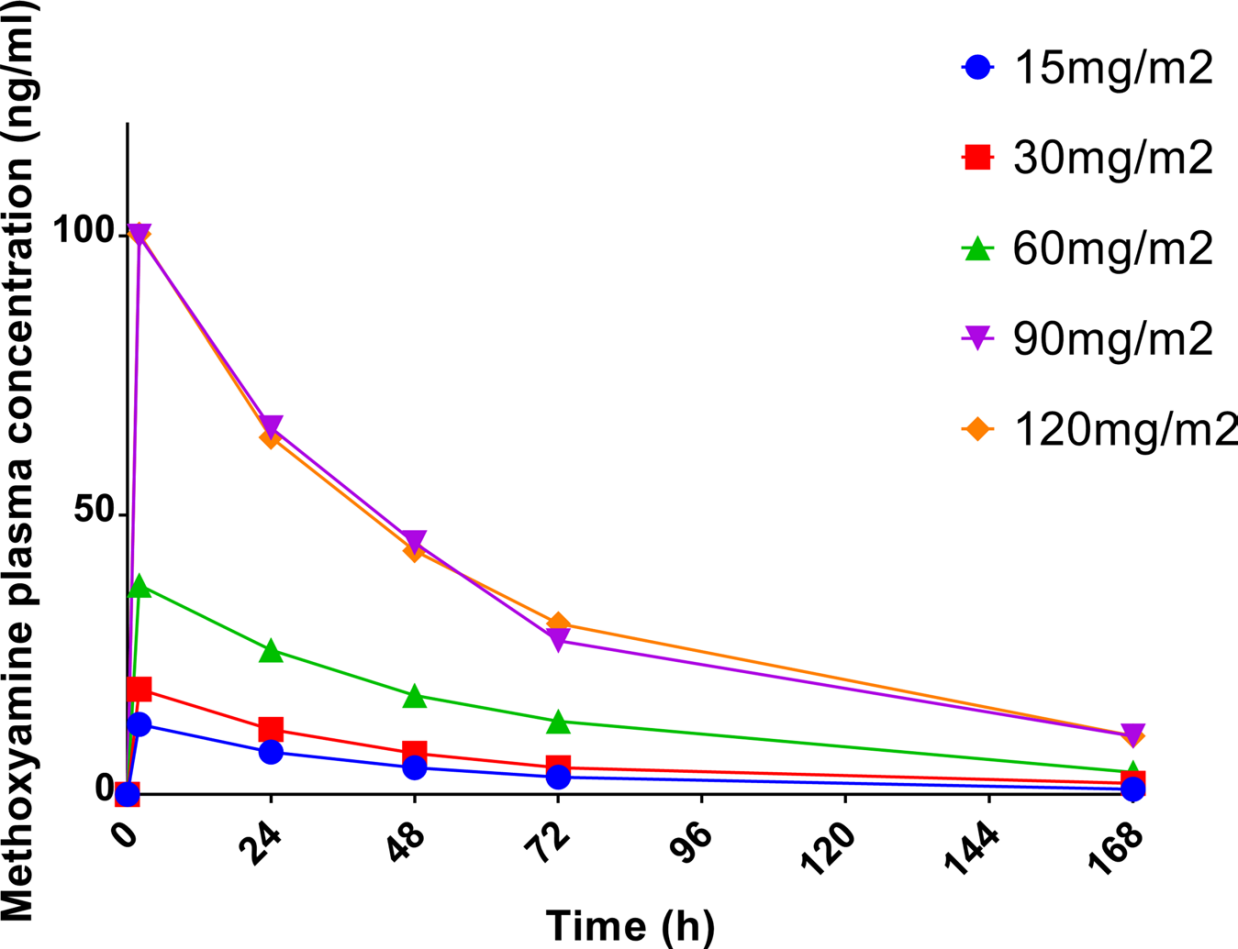
Average methoxyamine concentration in plasma over time after a single administration

The results of pharmacokinetic analyses are presented in Table 2. The half-life of methoxyamine ranged from 27.8 to 68.5 hours; median half-life was 40.1 hours; there was no statistically significant difference in the average half-life between dose levels. Linear regression analysis demonstrated a statistically significant correlation between methoxyamine dose and area under the curve (AUC) of plasma concentration (*p* < 0.0001; R^2^ = 0.67), as well as maximum plasma concentration (C_max_) (*p* < 0.0001; R^2^ = 0.73). Figure 1 illustrates the effect of methoxyamine dose on its plasma concentration over time, suggesting exponential decrease over time, compatible with first order elimination kinetics. Modeling with linear regression analysis of the natural logarithm of methoxyamine concentration (Ln[MX]) and time showed statistically significant correlation at each dose level (R^2^ = 0.901, 0.744, 0.748, 0.801 and 0.785 for 15, 30, 60, 90 and 120mg/m^2^ dose levels, respectively; *p* < 0.0001 for all correlations), confirming first order kinetics for methoxyamine.

**Table 2:** Pharmacokinetic parameters of methoxyamine

Dose	Half Life, mean (± SEM)	Cmax, mean (± SEM)	AUC, mean (± SEM)	Clearance, mean (± SEM)
**(mg/m^2^)**	**(h)**	**(ng/mL)**	**(h^ng/mL^)**	**(mL/h/m^2^)**
15	40.9 (± 7.0)	12.6 (± 1.3)	691.6 (± 44.8)	22062.0 (± 1445.4)
30	41.6 (± 8.9)	18.8 (± 5.4)	1044.4 (± 301.3)	34241.2 (± 9978.7)
60	44.9 (± 3.1)	37.5 (± 8.3)	2502.4 (± 816.3)	29014.3 (± 7954.4)
90	46.0 (± 5.9)	100.0 (± 21.3)	6560.6 (± 1560.1)	16035.0 (± 4895.5)
120	45.0 (± 5.2)	100.4 (± 13.8)	6358.2 (± 1042.2)	22180.8 (± 4371.2)

AUC: area under the curve of plasma concentration; Cmax: maximum plasma concentration.

### Pharmacodynamic studies – comet assay

Comet tail length presented a statistically significant increase from baseline measured at 24, 27, 48, 72 and 96 hours after treatment was started (*p* < 0.0001). There was no difference between comet tail length measured at baseline and 2 hours after first dose of fludarabine. There was no statistically significant difference in comet tail length between methoxyamine dose levels.

Linear regression analysis showed the change in WBC number after 1 week of treatment presented a statistically significant correlation with comet tail length at baseline (*p =* 0.029), 2 (*p =* 0.038), 24 (*p =* 0.030) and 27 (*p =* 0.032) hours after treatment. Correlation with later time points (48, 72 and 96 hours) was not statistically significant.

In patients with CLL and circulating malignant lymphocytes (*n =* 7), the average comet tail length increased 2.25 times over the first week of therapy (95% CI 0.81–3.68). The median absolute lymphocyte count decreased from 19.2 × 10^9^ lymphocytes/L (range 3.94–182.7) at baseline to 2.2 × 10^9^ lymphocytes/L (range 0.37–137.2) at day 7 (Figure 4 and Supplementary Table 1). Linear regression showed a statistically significant correlation between the percentage of decrease in circulating lymphocytes after 7 days and comet tail length at 24 (R^2^ = 0.93, *p =* 0.0004), 48 (R^2^ = 0.84, *p =* 0.004), 72 (R^2^ = 0.86, *p =* 0.003), and 96 (R^2^ = 0.72, *p =* 0.02) hours.

### Safety

Hematologic toxicity was common, including anemia (*n =* 20, 100%), lymphopenia (*n =* 16, 80%), neutropenia (*n =* 15, 75%) and thrombocytopenia (*n =* 13, 65%). The most common non-hematologic toxicities included fatigue (*n =* 17, 85%), nausea (*n =* 13, 65%), hypocalcemia (*n =* 11, 55%), anorexia (*n =* 10, 50%) and paresthesias (*n =* 10, 50%). Regard**i**ng grade ≥ 3 AEs, the most common (≥ 10% of pat**i**ents) grade 3 or 4 AEs observed were hematologic, including lymphopenia (*n =* 14, 70%), neutropen**i**a (*n =* 12, 60%), anemia (*n =* 8, 40%) and thrombocytopenia (*n =* 5, 25%). Comparison of the incidence of grade ≥ 3 adverse events between patients who received up to three cycles of treatment vs. those that received more than three cycles showed there was only a statistically significant difference in the incidence of leukopenia (*p =* 0.03), with no difference observed in the incidence of other hematologic toxicities.

Infection was the only common grade 3–4 non-hematologic toxicity (*n =* 3, 15%). Six patients presented a decrease in haptoglobin without clinical evidence of autoimmune hemolytic anemia or increases in plasma bilirubin and lactate dehydrogenase. All grade ≥ 3 AEs and all AEs occurr**i**ng **i**n more than one pat**i**ent are summar**i**zed in Table 3.

**Table 3:** Adverse events in subjects treated with fludarabine and methoxyamine

Toxicity	All Grades (%)		Grade 3 – 4 (%)	
Hematologic	*N*	%	*N*	%
Leukopenia	17	85	10	50
Neutropenia	15	75	12	60
Anemia	20	100	8	40
Thrombocytopenia	13	65	5	25
Lymphopenia	16	80	14	70
Gastrointestinal				
Anorexia	10	50	0	0
Constipation	9	45	0	0
Diarrhea	5	25	1	5
Nausea	13	65	0	0
Vomiting	5	25	0	0
Neurologic				
Insomnia	7	35	1	5
Dizziness	2	10	0	0
Paresthesias	10	50	0	0
Pain	8	40	1	5
Respiratory				
Cough	2	10	0	0
Dyspnea	7	35	0	0
General				
Fatigue	17	85	0	0
Cutaneous/Dermatologic				
Alopecia	3	15	0	0
Erythema	2	10	0	0
Pruritus	2	10	0	0
Infectious				
Fever	6	30	1	5
Infection	5	25	3	15
Cardiovascular				
Edema	3	15	0	0
Laboratory				
Hyperbilirubinemia	8	40	0	0
Hypokalemia	3	15	0	0
Hyperuricemia without TLS	3	15	1	5
Hypoalbuminemia	7	35	1	5
Hyponatremia	8	40	0	0
Hypocalcemia	11	55	0	0
Transaminase elevation	5	25	1	5
Creatinine elevation	5	25	0	0
Haptoglobin decrease	6	30	0	0

### Efficacy and survival

**Figure 2:**
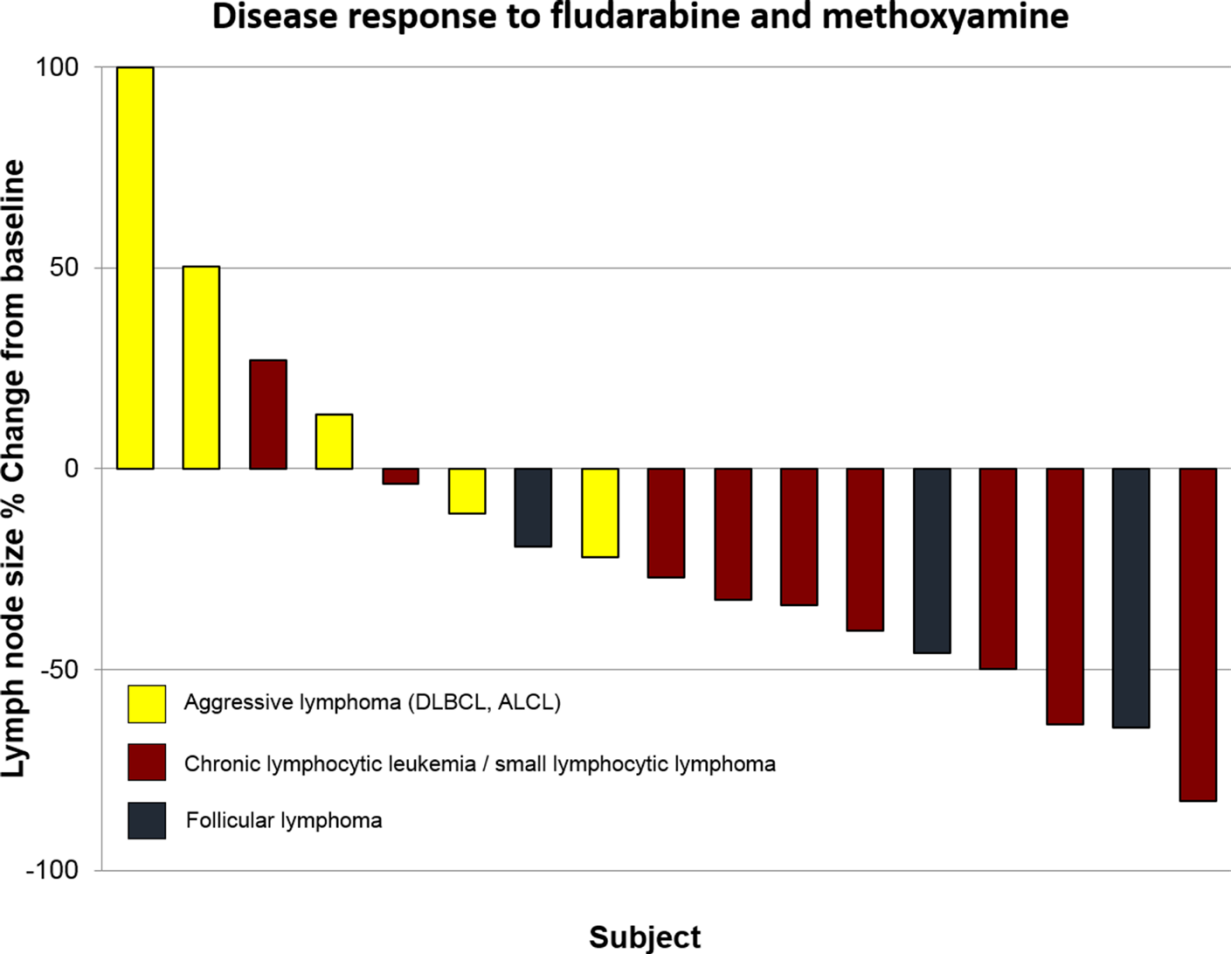
Waterfall plots of best overall response according to response criteria in 17 patients with measurable disease (patients excluded include one subject with CLL and no measurable lymph nodes and two subjects with plasma cell myeloma) (ALCL: anaplastic large cell lymphoma; DLBCL: diffuse large B cell lymphoma; MCL: mantle cell lymphoma)

Four patients (20%) achieved partial response (CLL/SLL = 3, follicular lymphoma = 1), and 8 patients (40%) exhibited stable disease (CLL/SLL = 6, follicular lymphoma = 2, DLBCL = 1) (Figure 2). Six CLL/SLL subjects had lymphocyte counts over 5 × 10^9^ lymphocytes/L and 5 presented normalization of lymphocytosis, with a median decrease of 95% in the first 7 days of therapy (range 29 – 99%, median baseline lymphocyte count = 72 × 10^9^ lymphocytes/L; median lymphocyte count on day 8 = 1.75 × 10^9^ lymphocytes/L).

Two subjects (CLL, follicular lymphoma) proceeded to reduced intensity allogeneic stem cell transplant and have not relapsed. One subject received maintenance rituximab for two years and has not progressed. All remaining subjects have either progressed (*n =* 6) or died (*n =* 11).

**Figure 3:**
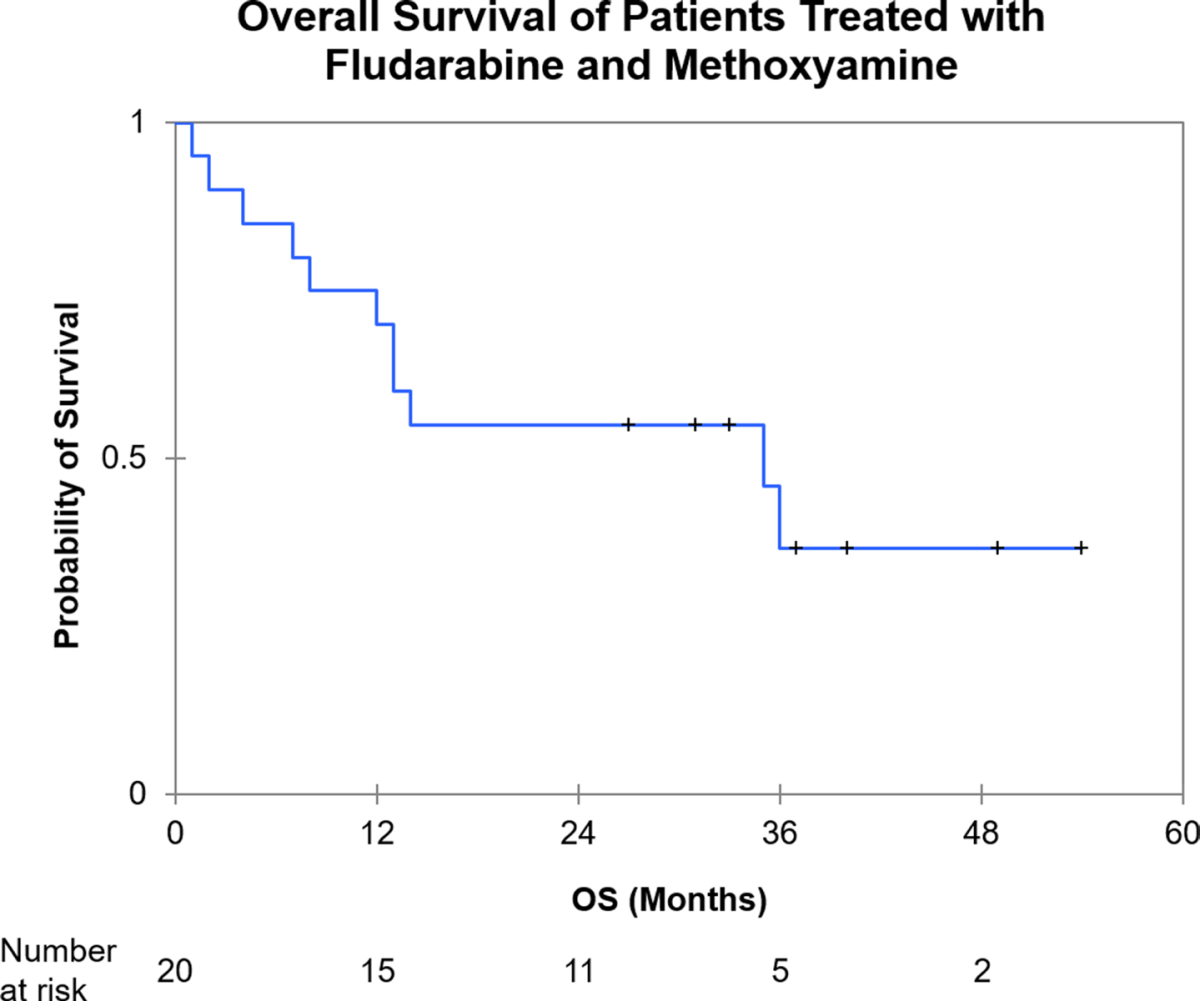
Kaplan Meier analysis of overall survival from treatment initiation (OS: Overall Survival).

After a median follow up of 27 months, 2–year OS was 55% (95% CI 33–77%) (Figure 3). For patients who achieved at least stable disease, 2 - year OS estimate is 92% (95% CI 76–100%) with a median time to next therapy of 9 months (95% CI 2–28).

**Figure 4:**
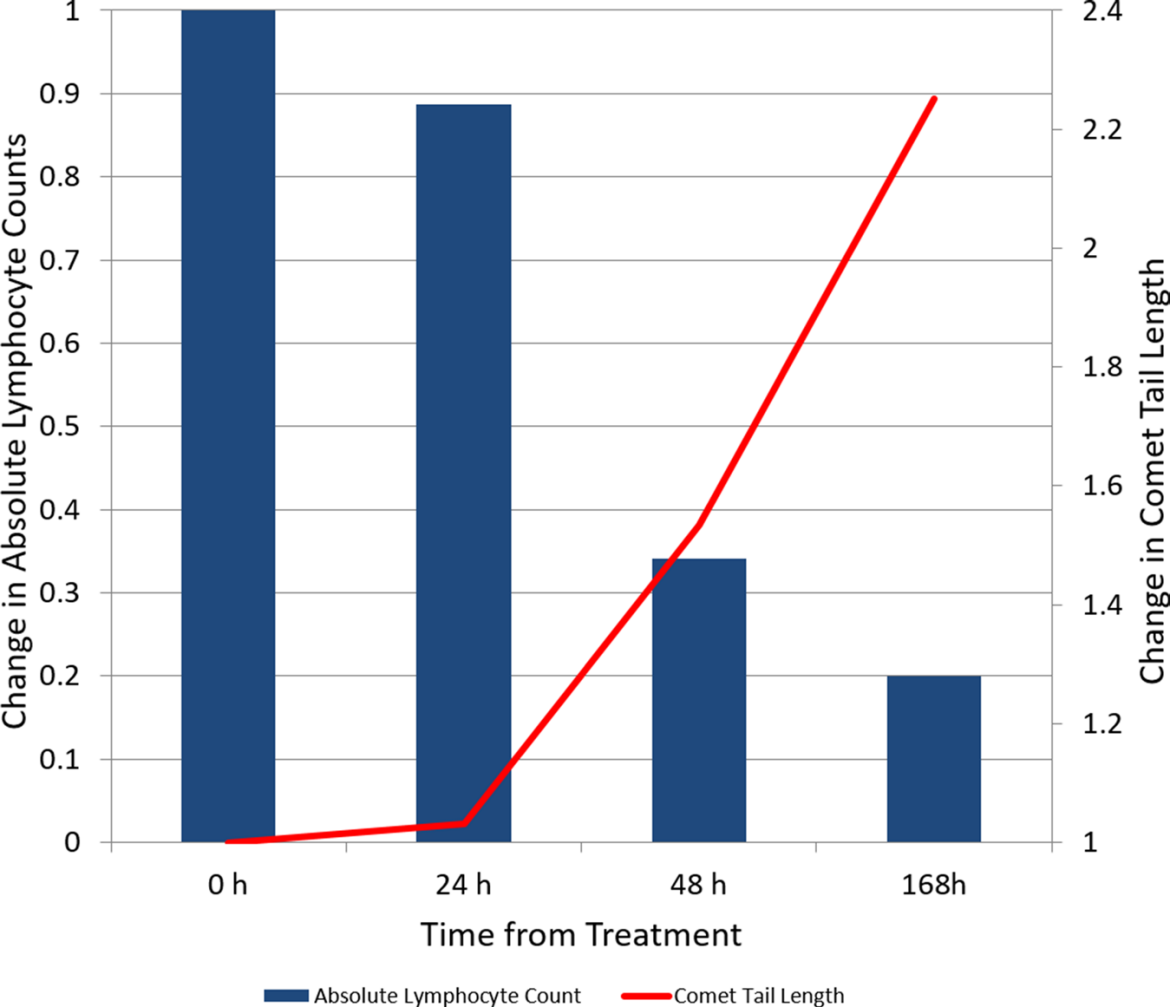
Change over time in absolute lymphocyte count and comet tail length in subjects diagnosed with chronic lymphocytic leukemia with peripheral blood lymphocytosis (*n* = 7)

## DISCUSSION

The BER system represents a potential mechanism of resistance to fludarabine. Methoxyamine is a first in class inhibitor of BER. The results of this phase I trial demonstrate that this drug can be safely administered in combination with fludarabine. There were no dose limiting toxicities and the maximum tolerated dose was not reached.

*In vitro* and animal studies suggest that the addition of methoxyamine to fludarabine increases the antitumor effect of the latter without added cytotoxicity to normal hematopoietic cells [24]. In accordance with these preclinical observations, the rate and severity of neutropenia (grade ≥ 3 = 60%) and thrombocytopenia (grade ≥ 3 = 25%) observed in our study were comparable to those previously reported for fludarabine. In a phase I–II study of single agent fludarabine for relapsed/refractory CLL [31], the rates of grade ≥ 3 neutropenia and thrombocytopenia were 56% and 25%, respectively. Leiby and colleagues reported the results of a phase I study of fludarabine monotherapy for subjects with relapsed non-Hodgkin lymphoma [32]; grade 3–4 leukopenia occurred in 4 of 21 subjects and 2 cases of grade 3 thrombocytopenia were observed. Fludarabine was given in continuous infusion, with a smaller total dose per cycle than in our study (initial loading dose of 20mg/m^2^ followed by continuous infusion of 30 mg/m^2^/day for 48 hours). Several randomized, controlled studies compared fludarabine with alkylating agents or combination chemotherapy [1, 6, 33]. In these trials, hematologic toxicity was common (grade ≥ 3 neutropenia: 19–38%; grade ≥ 3 thrombocytopenia: 13–15%; grade ≥ 3 anemia: 7–18%). The incidence and severity of anemia observed in our study was higher than that observed with first – line fludarabine monotherapy; this could be secondary to extensive prior therapy in our patient population, but could also be secondary to red blood cell membrane damage by methoxyamine, causing low-grade hemolysis and providing an explanation for decreases in haptoglobin observed in 6 subjects. Grade 3 anemia was observed in previous phase I studies of methoxyamine in combination with pemetrexed [34] or temozolomide [35]. Animal toxicology studies observed extravascular hemolysis at methoxyamine doses 20 times higher than those required for efficacy. Fludarabine has known cumulative hematologic toxicity [36] and it is possible that methoxyamine increases marginally the incidence of this group adverse events. While only 11 of 20 patients received at least three cycles of therapy, we observed that patients who received more than three cycles of the combination therapy had a higher incidence of leukopenia. Treatment duration did not increase the incidence of other hematologic toxicities, again suggesting that the addition of methoxyamine to fludarabine results in limited additional toxicity, with most of the hematologic adverse events manifesting in the first cycles of treatment. In our study, as in previously reported phase I studies of methoxyamine combinations, severe non-hematologic toxicities were rare and not different from reported for single agent fludarabine. Based on these observations and without clear increase in the incidence of toxicity with increasing dose, we concluded 120 mg/m2 as the recommended phase II dose of methoxyamine when studied in combination with fludarabine.

Previous studies have shown that addition of methoxyamine to fludarabine increased DNA double-strand breaks [24]. In our study, we observed cumulative increases in comet tail length throughout the first week of therapy, indicating progressive DNA damage. The observation of a correlation between decreases in circulating malignant lymphocytes and comet tail length highlights the relevance of DNA double-strand break measurements as a surrogate marker of the antineoplastic effect of methoxyamine and fludarabine.

As expected, responses were observed in subjects with indolent lymphoid malignancies, including three patients with CLL and one with FL. Among partial responders, two subjects had received prior fludarabine. Patients who achieved at least stable disease included only subjects with lymphoid malignancies. Both plasma cell myeloma subjects presented progressive disease after 2 cycles of therapy, suggesting methoxyamine did not overcome the absence of antimyeloma activity of fludarabine [37]. The concentration of responses and stable disease in cases with indolent lymphoma, and the rapid normalization of lymphocytosis in CLL patients reflects the selective lymphotoxicity of the combination.

Previous studies have demonstrated the safety of methoxyamine when combined with the antimetabolite pemetrexed in solid tumors [38]; phase II studies are evaluating the efficacy of the combination of methoxyamine with pemetrexed, cisplatin and radiation for treatment of non-small cell lung cancer (ClinicalTrials.gov NCT02535325 and NCT02535312). A phase I study investigating the combination of methoxyamine and the alkylator temozolomide for treatment of solid tumors and CNS malignancies has recently been completed [35]. Our present study shows methoxyamine can be safely combined with yet an additional class of antineoplastic agents. Additional *in vitro* studies are evaluating the combination of methoxyamine with other purine and pyrimidine analogs as well as bendamustine, an alkylating agent with purine analog – like properties used frequently for treatment of indolent lymphoid malignancies [39].

Fludarabine remains a central component of reduced intensity conditioning regimens for hematopoietic progenitor cell transplant. These regimens have more limited antitumor activity, and are primarily designed to be immunosuppressive, primarily through lymphodepletion, relying on the graft to eradicate the underlying neoplasm. Studies are planned to investigate the addition of methoxyamine to fludarabine – containing conditioning regimens, hypothesizing that the combination may result both in increased lymphodepletion as well as improved antineoplastic activity. Additional studies are planned to investigate the use of methoxyamine with alkylating agents used in stem cell transplant conditioning regimens, melphalan and busulfan.

With the introduction of new signaling inhibitors, the role of fludarabine in the treatment of indolent lymphomas and especially CLL has come into question [40, 41]. However, regimens containing this drug have excellent activity against CLL and remain relevant as preferred first line therapy for certain patient subsets [42]. Our study demonstrates that the addition of methoxyamine is safe and well tolerated and has activity against lymphoid malignancies, including subjects previously treated with fludarabine. The potential of methoxyamine to increase the antineoplastic activity of fludarabine with limited additional toxicity should be evaluated in further studies, both as a means of achieving higher antineoplastic activity and as a dose – sparing strategy.

Enhanced fludarabine activity may broaden its use in lymphoid malignancies as well as in stem cell transplant conditioning regimens, where increase activity of reduced intensity regimens can result in improved disease control.

## MATERIALS AND METHODS

### Study population

Eligible patients were adults older than 18 years of age with hematologic malignancies (excluding acute myeloid leukemia), relapsed after at least one line of therapy (no maximum), Eastern Cooperative Oncology Group performance status ≤ 2, adequate organ and bone marrow function (creatinine < 1.5 mg/dl or creatinine clearance > 60 ml/min/1.72 m^2^; total bilirubin < 1.5 mg/dl, AST < 2.5 times upper limit of normal; absolute neutrophil count > 750; absolute neutrophil count > 750/μl; platelets > 50,000/μl and hemoglobin > 9.0 g/dl). Patients who had progression after fludarabine therapy were eligible. Patients were excluded if they had received treatment within 3 weeks before starting treatment, if they had a history of uncontrolled heart failure (NYHA class III or IV), CNS or leptomeningeal involvement by disease, or if they had known HIV, hepatitis B or C infection.

The study protocol was approved by the institutional review board of University Hospitals Cleveland Medical Center. All patients gave written informed consent. The study was conducted according to good clinical practice and the declaration of Helsinki. It is registered at ClinicalTrials.gov (NCT01658319).

### Study design

This was a single center, open label, dose escalation trial designed to assess the safety and pharmacokinetics of methoxyamine when given in combination with fludarabine. Treatment cycles lasted 28 days. Fludarabine, 25 mg/m^2^ was given intravenously daily from day 1 through 5; methoxyamine was given intravenously on day 2 of cycle 1 to facilitate pharmacokinetic analysis, and then on day 1 of subsequent cycles. Methoxyamine dose escalation was done in 3 + 3 design, in five dose levels between 15 and 120 mg/m^2^. For further assessment of safety endpoints, the final cohort (120 mg/m^2^) was expanded to a total of 6 subjects.

### Study assessments

Adverse events were graded for severity according to the National Cancer Institute Common Terminology Criteria for Adverse Events (version 4.0). Dose limiting toxicities were defined as those occurring during the first cycle of therapy and included: grade ≥ 3 non-hematologic toxicities (with the exception of alopecia); grade 4 neutropenia lasting 14 or more days; febrile neutropenia (grade 4 neutropenia with fever > 38.5°C); neutropenic infection (grade ≥ 3 neutropenia with grade ≥ 3 infection); grade ≥ 3 hemolysis and grade ≥ 4 thrombocytopenia (or grade ≥ thrombocytopenia with grade ≥ 3 hemorrhage).

Baseline tumor measurements included complete blood count, bone marrow aspirate and biopsy and computed tomography (CT) imaging studies. Positron emission tomography imaging was done in patients with lymphomas known to be FDG avid, as delineated by Cheson and colleagues [43]. Serum and urine protein electrophoresis were done in plasma cell myeloma patients. Restaging laboratory and imaging studies were done every 8 weeks and at the end of treatment. The overall response rate was assessed by the investigator based on disease specific criteria [43–45]. Patients were observed for up to 1 year or until disease progression, death, or initiation of new anticancer therapy, whichever occurred first.

### Pharmacokinetic studies

Researchers from the Case Comprehensive Cancer Center developed and validated a liquid chromatography tandem mass spectrometry (LC-MS/MS) method for determining methoxyamine concentration in human plasma samples [46]. Blood samples for pharmacokinetic analysis were collected before methoxyamine infusion and 2, 24, 48, 96 and 168 hours after infusion. The maximum plasma concentration (Cmax), elimination half-life, and area under the curve for methoxyamine concentration were determined. All pharmacokinetic studies were performed by the Translational Research and Pharmacology Core of the Case Comprehensive Cancer Center.

### DNA damage measurements by comet assay

Peripheral blood samples were obtained at baseline, 2 hours after treatment with fludarabine alone on day 1, immediately before and 2 hours after methoxyamine and fludarabine infusion on day 2 and immediately before infusion of fludarabine on days 3, 4 and 5.

Peripheral blood mononuclear cells were isolated by Ficoll density gradient centrifugation.

The single cell comet electrophoresis assay was performed using Comet Assay kit (Trevigen, Gaithersburg, MD). Approximately 5000 mononuclear cells (in 50 μl) were mixed with 250 μl of 1% low melting point agarose in 1X PBS at 37°C. The mixture (75 μl) was pipetted onto a Comet slide (Trevigen, Gaithersburg, MD) and allowed to solidify at 4°C. Slides were then immersed for 30 minutes in prechilled lysis buffer (2.5 mM sodium chloride, 100 mM EDTA pH 10, 10 mM Tris Base, 1% sodium lauryl sarcosinate, 0.01% Triton X-100) at 4°C. After lysis, slides were incubated for 20 minutes in alkaline solution (0.3M NaOH, 1mM EDTA) at room temperature, to allow DNA unwinding, followed by alkaline electrophoresis for the next 30 minutes. The Comet shape in individual mononuclear cells was visualized by staining with the Comet silver staining kit (Trevigen, Gaithersburg, MD). Fifty cells per sample were analyzed using Komet Analysis software (Andor Technology, Belfast, UK). Cellular DNA damage was expressed as the tail “length”, tail DNA content, and tail “moment”, a measure that combines the length of DNA migration and the relative DNA content therein [47]. All pharmacodynamic assays were performed by the Translational Research and Pharmacology Core of the Case Comprehensive Cancer Center.

### Statistical analysis

To determine whether increasing doses of methoxyamine affect drug clearance we examined the difference among methoxyamine elimination half-lives of each cohort of this study using one-way ANOVA. For the analysis of the Comet assay, two-way ANOVA (with Bonferroni correction for multiple comparisons) was used to compare the means of tail length values at different time points as well as the effect of methoxyamine dose on tail length at different time points. The correlations of tail length values with changes in WBC and absolute lymphocyte count were estimated using linear regression. All tests were two sided and *p*-values were considered statistically significant if lower than 0.05. Statistical analyses were performed using XLSTAT (Addinsoft, New York, NY) and SAS Software (SAS Institute Inc., Cary, NC).

## SUPPLEMENTARY MATERIALS TABLE



## Abbreviations

AP: Apurinic/apyrimidinic
APE: Apurinic/apyrimidinic site endonuclease
BER: Base excision repair pathway
CLL: Chronic lymphocytic leukemia
Cmax: Maximum plasma concentration
CT: Computed tomography
DLBCL: Diffuse large B cell lymphoma
F-ara-ATP: Fludarabine triphosphate
FDG: Fludeoxyglucose (^18^F)
FL: Follicular lymphoma
LC-MS/MS: Liquid chromatography tandem mass spectrometry
NER: Nucleotide excision repair pathway
OS: Overall survival
PFS: Progression free survival
SLL: Small lymphocytic lymphoma
WBC: White blood cell
