# Supplementary material for: Phase I clinical trial of the base excision repair inhibitor methoxyamine in combination with fludarabine for patients with advanced hematologic malignancies
Source: Oncotarget. 2017 Aug 9;8(45):79864–75. doi: 10.18632/oncotarget.20094 (PMC5668101; doi:10.18632/oncotarget.20094)
Supplement: Supplementary file 1 [file oncotarget-08-79864-s001.pdf]

Phase I clinical trial of the base excision repair inhibitor methoxyamine in combination with fludarabine for patients with advanced hematologic malignancies

SUPPLEMENTARY MATERIALS

Supplementary Table 1: Changes in subjects with circulating malignant lymphocytes: Clinical laboratory studies, pharmacodynamic results, response and pharmacokinetic results

	Baseline	24 hours1	48 hours	168 hours	Response	Change in lymph node size at best response	MX dose level (mg/m^2)	MX half-life (hours)	MX Cmax (ng/mL)	MX AUC (h*ng/mL)
Patient 8										
ALC	19260	16580	4280	2290	SD	–33	30	38.23	29.6	1601.92
Comet Tail Length (n, SEM)	12.89 (94, 1.56)	13.83 (127,1.46)	31.75 (93,1.85)	46.93 (70, 2.02)						
Patient 9										
ALC	59930	51600	3660	2900	PD	–34	60	49.5	21.9	1425.88
Comet Tail Length (n, SEM)	5.22 (126,0.84)	13.49 (94,1.2)	16.72 (117,1.06)	20.46 (119,1.37)						
Patient 10										
ALC	84240	76190	21420	6230	PR	–50	60	38.92	40.6	1977.4
Comet Tail Length (n, SEM)	6.77 (49, 2.33)	2.67 (150,0.45)	3.25 (131,0.54)	2.51 (108,0.6)						
Patient 15										
ALC	182750	184800	158860	137590	SD	NM[2]	120	33.54	159	6998.87
Comet Tail Length (n, SEM)	86.06 (88,2.64)	87.96 (100,2.36)	84.07 (107,2.52)	87.01 (86,2.61)						
Patient 17										
ALC	3940	2680	140	370	SD	–27	120	68.47	113	10268.35
Comet Tail Length (n, SEM)	5.49 (92,1.04)	9.5 (137,1.4)	10.27 (119,1.25)	22.07 (53,3.51)						
Patient 18										
ALC	4300	2140	500	850	PD	27	120	47.56	97.1	6126.17
Comet Tail Length (n, SEM)	1.73 (81,0.4)	1.35 (44,0.3)	1.25 (48,0.18)	2.38 (75,1.24)						
Patient 19										
ALC	6940	8430	6310	1510	PR	–82	120	37.73	58.9	2895.84
Comet Tail Length (n, SEM)	8.65 (117,1.7)	8.7 (91,1.8)	8.7 (91,1.8)	12.12 (107,1.57)						

One hour after methoxyamine infusion.
Non measured disease, subject only presented lymphocytosis.
ALC: absolute lymphocyte count; AUC: area under the curve; Cmax: maximum concentration; MX: methoxyamine; NM: non measured disease; PD: progressive disease; PR: partial remission; SD: stable disease; SEM: standard error of the mean.
